# Supplementary material for: Saccharomyces boulardii (CNCM I-745) improves intestinal damage in sepsis by remodeling intestinal flora structure
Source: Front Cell Infect Microbiol. 2025 Jul 31;15:1602792. doi: 10.3389/fcimb.2025.1602792 (PMC12350329; doi:10.3389/fcimb.2025.1602792)
Supplement: Supplementary file 1 [file DataSheet1.zip › summary/summary/7_Differential_analysis/2_LEfSe_result/Group_AW4_vs_AW0_0.05_3.0/LEfSe_Group_AW4_vs_AW0.g.barplot.pdf]

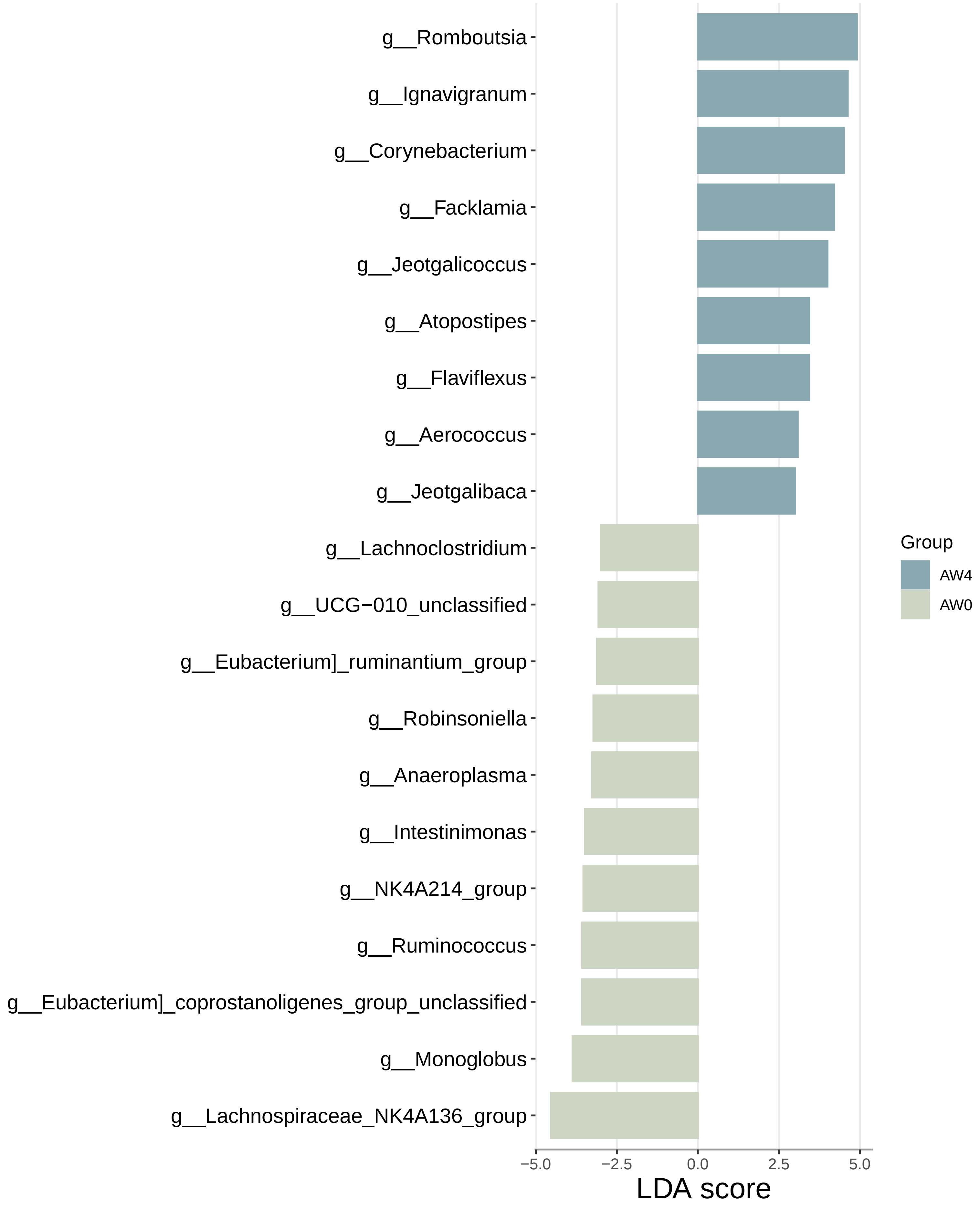
g__Romboutsia
g__Ignavigranum
g__Corynebacterium
g__Facklamia
g__Jeotgalicoccus
g__Atopostipes
g__Flaviflexus
g__Aerococcus
g__Jeotgalibaca
g__Lachnoclostridium
g__UCG−010_unclassified
g__Eubacterium]_ruminantium_group
g__Robinsoniella
g__Anaeroplasma
g__Intestinimonas
g__NK4A214_group
g__Ruminococcus
g__Eubacterium]_coprostanoligenes_group_unclassified
g__Monoglobus
g__Lachnospiraceae_NK4A136_group
−5.0
−2.5
0.0
2.5
5.0
LDA score
Group
AW4
AW0
